# Mending a Broken Heart: Treatment of Stress-Induced Heart Failure after Solid Organ Transplantation

**DOI:** 10.1155/2018/9739236

**Published:** 2018-02-18

**Authors:** N. Thao Galván, Kayla Kumm, Michael Kueht, Cindy P. Ha, Dor Yoeli, Ronald T. Cotton, Abbas Rana, Christine A. O'Mahony, Glenn Halff, John A. Goss

**Affiliations:** ^1^Division of Abdominal Transplantation and Hepatobiliary Surgery, Baylor College of Medicine, 6620 Main Street, Suite 1425, Houston, TX 77030, USA; ^2^University of Texas Health Science Center at San Antonio, UT Transplant Center, 4502 Medical Drive, San Antonio, TX 78229, USA

## Abstract

Stress-induced heart failure, also known as Broken Heart Syndrome or Takotsubo Syndrome, is a phenomenon characterized as rare but well described in the literature, with increasing incidence. While more commonly associated with postmenopausal women with psychiatric disorders, this entity is found in the postoperative patient. The nonischemic cardiogenic shock manifests as biventricular failure with significant decreases in ejection fraction and cardiac function. In a review of over 3000 kidney and liver transplantations over the course of 17 years within two transplant centers, we describe a series of 7 patients with Takotsubo Syndrome after solid organ transplantation. Furthermore, we describe a novel approach of successfully treating the transient, though potentially fatal, cardiogenic shock with a percutaneous ventricular assistance device in two liver transplant patients, while treating one kidney transplant patient medically and the remaining four liver transplant patients with an intra-aortic balloon pump. We describe our experience with Takotsubo's Syndrome and compare the three modalities of treatment and cardiac augmentation. Our series is novel in introducing the percutaneous ventricular assist device as a more minimally invasive intervention in treating nonischemic heart failure in the solid organ transplant patient, while serving as a comprehensive overview of treatment modalities for stress-induced heart failure.

## 1. Introduction

Stress-induced heart failure, also known as Broken Heart Syndrome or Takotsubo Syndrome, is a phenomenon first reported in 1990. Not an ischemic phenomenon, it is characterized by transient left ventricular (LV) apical akinesis with hypercontraction of the basal wall creating a ballooning appearance of the heart. It resembles the Japanese octopus trap giving it its moniker, Takotsubo. While previously believed to be a rare syndrome, the literature has a few larger volume series and registries that have been able to better characterize it, with evidence of a 19-fold increase in incidence between 2006 and 2012, suggesting that it is in fact underrecognized and more prevalent than previously understood [1–5]. While most commonly described in elderly, postmenopausal female patients with a strong association of neurologic or psychiatric disease, it has also been recognized in the postoperative patient. The hypothesized pathophysiology is a hyperadrenergic load that leads to a transient ventricular dyskinesis, unaccompanied by occlusive coronary artery disease (CAD) [3, 6, 7].

Clinical manifestations include ST elevation, depression, or T-wave changes, prolonged Qt interval, and mild increases in cardiac enzymes [3, 7]. Ninety percent of the time, complete resolution of apical wall motion abnormality and depressed LV systolic function occurs without an associated adverse event [7, 8]. While stress-induced heart failure is often self-limited and resolves with medical support alone, mechanical ventricular support is occasionally necessary to avoid death, especially in the presence of cardiogenic shock [9]. It is as deadly as an acute coronary syndrome with significant morbidity and mortality ranging from 5.8 to 27% and thus should be addressed with the same gravity [2, 7]. In a Swedish national registry, when Takotsubo is identified with physical triggers (as surgery), acute neurologic or psychiatric disease, an admission troponin measurement greater than 10 times the normal value, and an EF of less than 45%, an association for poor outcome was found [10]. As average survival after transplantation improves and centers, in fact, have increasing oversight in regard to their outcomes, the onus is on transplant centers to ensure maximal survival not only for the sake of the patients, but also for the sake of their transplant programs [11]. Furthermore, emerging evidence suggests that while the condition may be self-limiting the majority of times, 81.4% of in-house mortality with Takotsubo Syndrome was associated with underlying critical illness, like liver transplantation, and in this case, males were more susceptible than females [5, 12]. There are currently two evidence-based guidelines for the management of patients with Takotsubo, and, for severe cases, they mention the use of intra-aortic balloon pumps, left ventricular assist devices, and extracorporeal membrane oxygenation as a bridge to recovery [3, 13].

We describe our experience with stress-induced heart failure after orthotopic liver transplantation (OLT) and kidney transplantation in two transplant centers. Currently case reports and a few case series describe treatment of this entity with medical management or intra-aortic balloon pump (IABP). Furthermore, there is one study to describe the use of a LVAD to support Takotsubo cardiomyopathy [25] (Table 1). We present the largest series yet, 7 surgical patients who suffered Takotsubo Syndrome after solid organ transplantation treated with three modalities: medical management, IABP, and mechanical flow support with a percutaneous ventricular assist device (pVAD). To our knowledge, this is the first to describe the successful use of a pVAD in the treatment of cardiogenic shock due to Takotsubo's Syndrome in liver transplant patients in the US.

## 2. Materials and Methods

We performed a retrospective review of 2,303 liver and 734 kidney transplantations performed between two centers over 15 years (1998–2015) and identified 7 cases of posttransplant acute nonischemic heart failure. This required extensive review of paper charts up until the 2000s when electronic medical records occurred at both centers, after which electronic charts were mined. Then electronic records were reviewed up to 2015. Specifically, patient cardiac catheterizations were reviewed, both preoperatively and postoperatively, and review of postoperative echocardiographies was reviewed to assess for signs of acute changes and signs of Takotsubo Syndrome. Once that was identified, the case was reviewed in toto. All cases of nonischemic heart failure occurred within 7 days of transplantation, with the first case identified in 2009. Each case is characterized by patient demographics including gender, age, associated comorbidities, and diagnosis in addition to pre- and postoperative cardiac studies. We highlight these cases and detail the novel use of the TandemHeart™ pVAD (Houston, TX) in two of these cases.

### 2.1. Medical Management

**Case 1 .**

**Figure 1:**
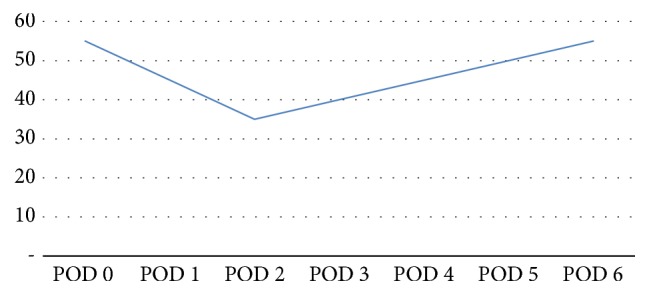
Ejection fraction with medical management after kidney transplant. Ejection fraction of Case 1 from postoperative day (POD) 0 over time.

A 45-year-old female with situs inversus, hypertension, hypothyroidism, and IgA nephropathy underwent living-related kidney transplantation. Preoperative transthoracic echocardiography (TTE) revealed preserved left ventricular ejection fraction (LVEF) of 55–60% but with impaired relaxation. Pulmonary arterial (PA) systolic pressures were 25–30 mmHg. Surgery was complicated by intraoperative hypotension requiring dopamine infusion as well as hypocalcemia secondary to intraoperative plasmapheresis, which was part of her immunosuppression induction protocol. She was extubated the same day. On postoperative day (POD) 2, she suffered a cardiac arrest after ventricular tachycardia, requiring 2 minutes of advanced cardiac life support (ACLS) and intubation. TTE revealed apical ballooning with severely decreased overall LV systolic function (LVEF 35%) consistent with Takotsubo Syndrome (TS). She maintained a normal serum creatinine of 0.8 mg/dL while being medically supported with vasopressors. By POD 4, the apical ballooning and the compromised EF were completely resolved. A repeat TTE on POD 6 showed normal systolic function (EF 55%, Figure 1) and normal chamber sizes. Cardiac catheterization was performed and proved no CAD. She was discharged on POD 9 and has since convalesced without further issue, with normal allograft function.

### 2.2. Intra-Aortic Balloon Pump

**Case 2 .**

A 54-year-old man with hypertension, hyperlipidemia, subclinical hypothyroidism, and alcoholic cirrhosis (Model for End Stage Liver (MELD) score 37) underwent OLT. Preoperative echocardiography and a nuclear stress test revealed normal heart function without evidence of ischemia. There were no intraoperative complications. Overnight, he became hypotensive with atrial fibrillation with rapid ventricular response. He converted successfully to a sinus rhythm with amiodarone and vasopressin was started for hypotension. TTE showed mildly dilated ventricles bilaterally with severely decreased biventricular systolic function, (EF < 20%) and trace mitral and tricuspid regurgitation, consistent with a Takotsubo Syndrome. Serial cardiac enzymes remained negative and his liver allograft continued to have normalizing liver function tests although he developed oliguric renal failure. Right and left heart catheterization confirmed poor ventricular function and no evidence of coronary disease and ischemia and an IABP was placed. Repeat TTE on POD 3 showed no significant change and continuous venovenous hemodialysis (CVVHD) was begun.

**Figure 2:**
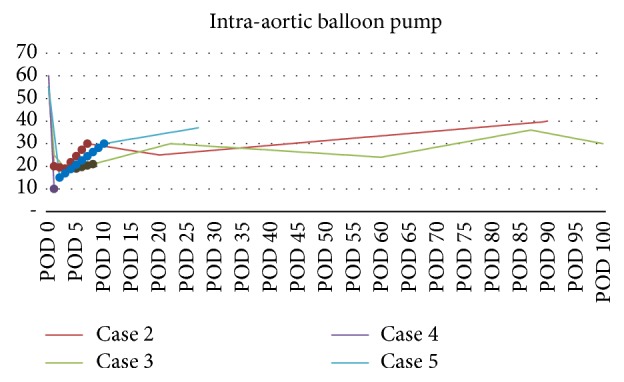
Ejection fraction after intra-aortic balloon pump after OLT. Ejection fraction of Cases 2–5 from POD 0 over time. Dotted portion of each line marks time of intra-aortic balloon pump use.

Echocardiogram POD 7 revealed no LV asymmetric wall motion abnormalities, mild-moderately dilated RV, and a moderate increase in systolic function (EF 30%); the IABP was removed. He was extubated on POD 9 and CVVHD was discontinued. On POD 20, TTE showed an EF of 25–35%, persistent global hypokinesis of LV, and elevated LV filling pressures. Beta blockade was begun; amiodarone was discontinued. The patient continued to improve and he was discharged on POD 30. Three months later, TTE showed an EF of 40% (Figure 2), normal LV filling pressures, and normal RV function. His liver allograft is functioning well.

**Case 3 .**

A 34-year-old male with sleep apnea, hypertension, and alcoholic cirrhosis (MELD 36) underwent OLT. Preoperative TTE showed normal functions (EF 55–60%). His surgery involved a large blood loss and the perioperative course was significant for severe volume overload in the context of acute kidney injury. On POD 1, TTE revealed systolic dysfunction with EF of 25–29%, severe global LV hypokinesis, and elevated PA pressures. By POD 3, TTE showed EF of <20%, severely decreased LV systolic function, severely enlarged right atrium, reduced RV systolic function, and severe mitral and tricuspid regurgitation. His midventricular wall motion abnormality was read as consistent with Takotsubo variant morphology. Systolic PA pressures were estimated at 50 mmHg. An IABP was placed POD 5, with milrinone and digoxin for inotropic support. The IABP was removed on POD 8 and, after inpatient rehabilitation, he was discharged on POD 22 with an EF of 30–34% and PAP 40–45 mmHg. Follow-up echocardiography continued to show compromised LV function with an EF ranging between 20 and 34%. This was exacerbated by difficulties with patient adherence to the medication regimen. Because EF never improved beyond 35% (Figure 2), a single-chamber ICD was placed 5 months postoperatively. His liver allograft is functioning well with normal liver function tests.

**Case 4 .**

57-year-old male with hepatitis C (HCV) and alcoholic cirrhosis (MELD 36) underwent uneventful OLT. Preoperative echo showed mild atrial and RV dilation, with mild diastolic dysfunction and LVEF > 60%; no preoperative cardiac catheterization was obtained. Intraoperative TEE was normal. On POD 1, he decompensated with hypoxia and dyspnea requiring intubation. TTE revealed EF of 10% (Figure 2) with global hypokinesis and cardiac catheterization revealed no significant CAD. An IABP was placed. Unfortunately, the patient passed from massive upper GI bleed while on a heparin infusion for IABP on POD 2.

*Patient 5.* A 46-year-old female with anxiety, depression, chronic renal insufficiency, and alcoholic cirrhosis (MELD 29) underwent OLT. She had a pacemaker for second-degree heart block and preoperative TTE revealed a mildly dilated left atrium with EF > 55%. Intraoperatively, there was a brief episode of unexplained hypotension (<5 minutes) with systolic pressures in the 50 s. After extubation on POD 2, she became tachypneic and oliguric, showing signs of heart failure. TTE revealed an EF of 15%, severe diastolic dysfunction, and akinesia of the apex with ballooning, basolateral-septal, mid, and apical-anterior wall segments in addition to severe tricuspid regurgitation and thus she was diagnosed with TS. An IABP was placed and supported the patient until removal on POD 10. TTE showed an improved EF at 30–35%, which was stable at 37% a week later (Figure 2). On POD 24, she was discharged in stable condition with a functioning liver allograft.

### 2.3. Percutaneous Ventricular Assist Device

**Case 6 .**

A 57-year-old male with HCV, hepatocellular carcinoma, alcoholic cirrhosis (MELD 26), and hepatopulmonary syndrome requiring home oxygen underwent an uneventful OLT. His heart history was significant only for a small asymptomatic patent foramen ovale. Preoperative TTE revealed a mildly dilated LV and left atrium with an EF of 60–65%. Pretransplant cardiac catheterization revealed no coronary lesions. He was extubated on POD 1 and progressed as expected until POD 7, when he became hypotensive with hypoxia and atrial fibrillation with rapid ventricular rate (RVR). TTE showed severe biventricular systolic dysfunction (EF < 20%) and severe biatrial enlargement, consistent with Takotsubo Syndrome. Transesophageal echocardiogram confirmed global hypokinesis; there was no evidence of thrombus. Electrocardiograms revealed no ischemia along a vascular distribution but the patient was too unstable for a cardiac catheterization. Vasopressin was begun and multiple attempts at cardioversion failed. On POD 9, atrial fibrillation with RVR continued along with worsening hypoxia, increasing transaminases, and worsening coagulation profiles. Considering the extent of cardiogenic shock and allograft dysfunction, a pVAD (TandemHeart) was placed and CVVHD was started. Additionally, he underwent plasmapheresis and IVIG infusion for possible rejection and cytokine storm. Prior to removal of the pVAD on POD 22, TTE revealed a modest improvement in the EF to 30–35% and resolution of the global hypokinesis, although RV function was still poor. Over the next several days, the patient continued to experience intermittent episodes of unsustained atrial fibrillation and ventricular tachycardia which resolved with amiodarone and beta blockade. TTE on POD 32 showed normal LV size with EF of 40–44%.

**Figure 3:**
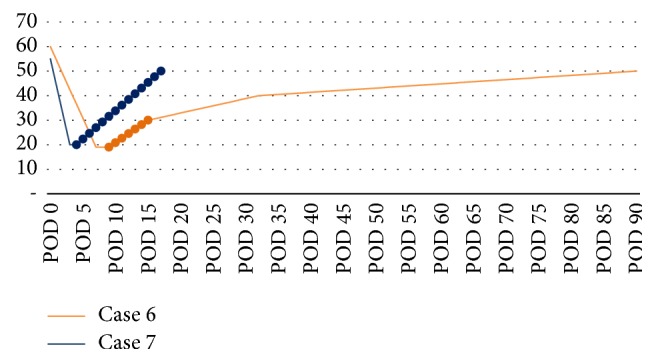
Ejection Fraction after percutaneous ventricular assist device after OLT. Ejection fraction of Cases 6 and 7 from POD 0 over time. Dotted portion of each line marks time of percutaneous ventricular assist device.

He was discharged on POD 56 with normal liver function tests and coagulation profiles and improved renal function. Follow-up TTE after 3 months confirmed further improvement of EF to near his pretransplant baseline, 50–55% (Figure 3).

**Case 7 .**

A 66-year-old male with autoimmune hemolytic anemia, chronic renal insufficiency, diabetes, hypertension, HCV, hepatocellular carcinoma, and alcoholic cirrhosis (MELD 23) underwent uneventful OLT. His cardiac history was significant for CAD for which he underwent coronary artery bypass 5 years earlier. Heart catheterization showed normal contractility with an occluded vein graft to a diagonal branch, but with retrograde filling. The distal LAD was occluded beyond the graft anastomosis. Preoperative TTE revealed normal LV function with EF of 55–60%. He was extubated on POD 1. On POD 3, he became hypotensive with hypoxia and acidosis in addition to having elevated transaminases. Echocardiography revealed LV apical ballooning with EF of 20–25%. On POD 4, coronary angiography revealed stable disease and a TandemHeart pVAD was inserted and his hypotension, acidosis, and hypoxia rapidly improved. Treatment was continued for 13 days with normalization of his liver enzymes. Prior to removal, TTE revealed a substantially improved EF of 50–55% and resolution of the apical akinesis (Figure 3). He was discharged on POD 58 and his allograft continues to function well.

## 3. Discussion

We report the successful use of a pVAD in the treatment of 2 liver transplant cases of postoperative nonischemic, stress-induced heart failure. Contrary to primary Takotsubo Syndrome where treatment is focused solely on the heart, management of secondary Takotsubo Syndrome as in the posttransplant patients described here must include postoperative care in addition to immunosuppression. Although the syndrome is characteristically transient, mortality is estimated between 10 and 27% and is higher in those who present with cardiogenic shock. In the immediate setting, the syndrome can lead to major adverse cardiac and cerebrovascular events at 9.9% per patient-year, with a morbidity of 5.6% [1, 7]. Major adverse cardiac and cardiovascular sequelae of this entity include acute stroke, cardiogenic shock, arrhythmia, thrombus formation, left ventricular outflow tract obstruction, pericarditis with effusion, and ventricular wall rupture [3, 8, 26–28]. In the critical posttransplant period, failure to rescue can result in severe morbidity, including allograft compromise, and death. To date, a limited number of case reports have described the use of IABPs to augment cardiac output in post-OLT heart failure, and they are listed in Table 1. Extracorporeal membrane oxygenation (ECMO) has been described on a few European case reports, and LVAD has been suggested as a rescue maneuver to support the low cardiac output as a bridge to recovery for high-risk Takotsubo, though not in transplant recipients [3, 13]. In our experience, a percutaneous VAD can be especially useful, and perhaps preferred, over the IABP after abdominal solid organ transplantation.

Our series is limited in that it is a retrospective review and dependent on review of copious paper and electronic charts with missing data, specifically data on pulmonary artery catheter measurements that helped to guide treatment. Having consistent data on pulmonary artery measurements would have strengthened the ability to inform treatment algorithms. Furthermore, 7 patients identified with posttransplant Takotsubo Syndrome are not many when culled from databases of over 3000 transplant patients. Our first patient was not identified until 2009, which suggests we may not have recognized this entity until later and it may be an underestimate of actual cases. This is consistent with a study that reviewed nationwide trends on TS between 2006 and 2012, and the reported incidence increased 19-fold during that time, perhaps related to improved recognition [5]. While the census is low, it may prove the point that failure to recognize this disease process means it is not as rare as previously believed and may in fact be more relevant to the transplant community. Assessing the role of exogenous catecholamines in this syndrome cannot be well-assessed in this series as our center's protocol mandates that all liver transplant recipients (if tolerated) receive empiric intraoperative dopamine infusions. Another limitation is that two of our patients did not have pre- or postoperative cardiac catheterizations, and while their echocardiograms, clinical courses, and biochemistries were convincing of the diagnosis of Takotsubo's Syndrome, it does not have the gold standard test to rule out an ischemic heart failure. It does bring up an important point, though, as in Case 7, that coexistence of coronary artery disease does not exclude Takotsubo's Syndrome [10].

**Figure 4:**
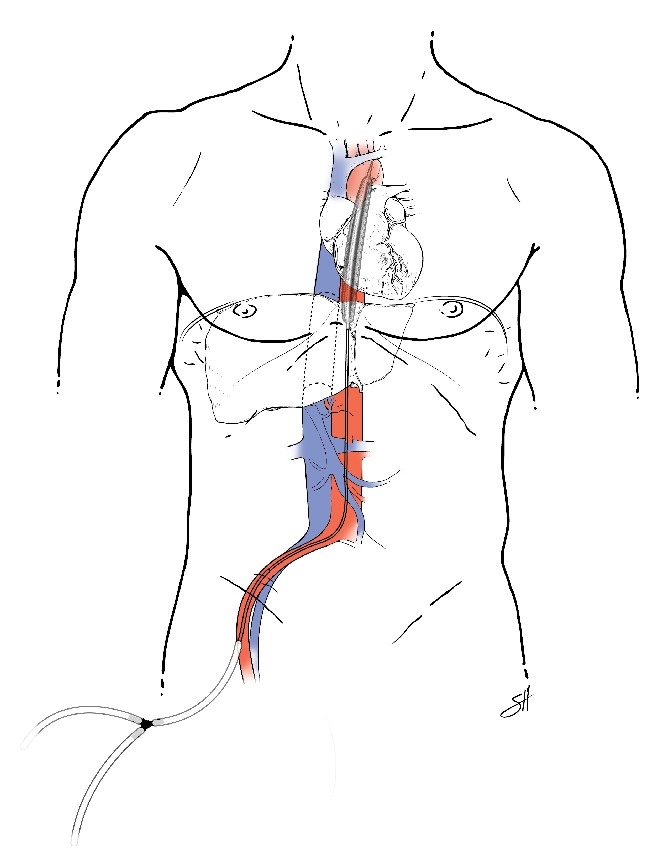
Intra-aortic balloon pump after OLT for Takotsubo Syndrome. An intra-aortic balloon pump inserted in the right femoral artery, traversing the aorta as it passes the celiac axis, in the setting of Takotsubo Syndrome after an orthotopic liver transplantation. Typical arterial access cannula size 8 Fr (2.7 mm).

**Figure 5:**
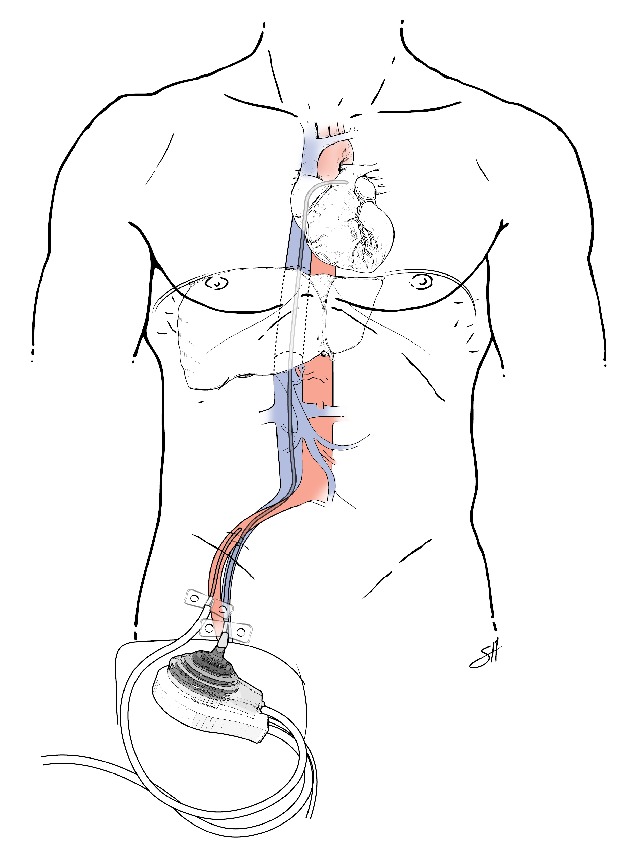
Percutaneous mechanical assist device after OLT for Takotsubo Syndrome. A percutaneous mechanical assist device, inserted in the right femoral artery and vein, in the setting of Takotsubo Syndrome after orthotopic liver transplantation. Typical venous access cannula size 21 Fr (7 mm). Typical arterial access cannula size 15 Fr (5 mm).

All 7 of our patients had cardiogenic shock with biventricular failure and significant decreases in EF and cardiac function. One patient was treated with medical management, 4 were treated with IABPs (Figure 4), and 2 were treated with pVADs (Figure 5). In the 2 patients rescued with a pVAD, mechanical support was continued for 13–15 days with eventual recovery of cardiac function to near pretransplant levels, and, notably, without any loss in allograft function. Five of the six OLT patients improved during the course of their hospitalizations and were discharged home with varying degrees of cardiac improvement upon follow-up.  Case 4, however, suffered death from GI bleeding due to the use of heparin infusion for the IABP on POD 2. This death brings up a salient limitation in the use of pVADs in the posttransplant and, in fact, postoperative patient. For all percutaneous assist devices, anticoagulation is necessary and indicated, and in the immediate postoperative setting, this presents a real limitation for the use of pVADs [29]. Most patients described had TS manifest within 48 hours of the perioperative period. However, if the patient has no evidence of bleeding and his cardiac shock leads to end organ dysfunction with benefits outweighing the risks, the utility of pVADs for treatment of Takotsubo's is evident.

It is noteworthy that the only patient who exhibited any shock liver was Case 6, who had his pVAD placed to support his hypoperfusion. While there is certainly no power in this study to support a causal relationship, the two patients who had a pVAD had near-complete resolution of their cardiac function (Figure 3) as compared to the four patients who had an IABP (Figure 2). This relationship deserves more study, especially considering the neutral data of the IABP-SHOCK II study [30]. The decision to use a pVAD over an IABP was because of the capabilities of our facilities and the fact that our patients were persistently unstable, with evidence of allograft dysfunction. Upon further review, IABP have been implicated in worsening the left ventricular outflow tract obstruction in Takotsubo's [3, 31, 32]. A European consensus study on Takotsubo's Syndrome recommended that if a patient with primary Takotsubo has cardiogenic shock with progressive multiorgan failure, it is prudent to consult for the use of ECMO or LVAD as a bridge to recovery [3, 33]. As it pertains to the case of secondary Takotsubo, as is for this series of cases, we have shown success with the use of percutaneous ventricular support. It is effective, minimally invasive, and temporary and requires no cardiopulmonary bypass. Nonetheless, the use of advanced mechanical circulatory support requires available expertise and carries its own set of risks including the potential need for a transseptal puncture and much larger sized cannulae for the vascular access.

In the context of altered vascular anatomy after liver or kidney transplantation, vascular compromise to the allograft and risks to the patient must be taken into special consideration when choosing a method of cardiac output augmentation. The pVAD is especially useful in conditions anticipated to be transient because of the ease of insertion and monitoring [34, 35]. Our one kidney transplant patient required medical support alone for resolution of her heart failure, but the theoretical benefits of pVAD over IABP would still apply for the unstable kidney transplant patient in cardiogenic shock. The pVAD avoids the need for cardiopulmonary bypass surgery for insertion and can be used for extended periods beyond those suggested for IABPs, making it ideal for patients in whom a major surgery is to be avoided [17]. Importantly, the use of IABP has been suggested to exacerbate mesenteric and renal ischemia by occlusion in a review of 106 patients, in a situation with already compromised perfusion, even with proper placement [36]. While the IABP augments diastolic perfusion in the descending aorta, the TandemHeart pVAD traverses the inferior vena cava (IVC) and terminates in the left atrium via a transseptal puncture (Figure 4). Its placement eliminates the potential occlusion of the celiac axis and supraceliac aorta; thus obstruction of the arterial vascular supply to the liver allograft is avoided with a pVAD (Figure 5). In the postoperative immunosuppressed LT patient, the risks to patient and graft are multiplied in cardiogenic shock [12]. Fortunately, if adequate cardiac support is provided in a timely manner, the patient can survive long enough for the condition to resolve.

Based on our experiences with liver transplant patients, a pVAD may serve as a life-saving bridge to cardiac recovery in the treatment of stress-induced heart failure. In our series of 7 surgical patients, we successfully used a TandemHeart pVAD to treat 2 cases of posttransplant cardiogenic shock. The pVAD provided a combination of ease of insertion and removal in the setting of severely compromised heart function. Its use may be anatomically and physiologically more forgiving to the new transplanted allograft in the setting of cardiogenic shock than IABPs as it avoids the potential of mesenteric aortic occlusion and yet allows for a longer duration of temporary support. In fact, IABPs are implicated in worsening left ventricular outflow obstruction, and recommendations against its use have emerged for Takotsubo Syndrome [13].

Review of the literature in combination with our experiences in liver transplant patients suggests that of those centers with the specialists and capabilities to allow for it, the pVAD may serve as a life-saving maneuver for cardiac and allograft recovery in the treatment of Takotsubo Syndrome within the transplant population. We believe the rarity but increasing incidence of this phenomenon dictates inclusion of all the modalities used at our centers and described in the literature, which allows the reader to understand the breadth of treatment modalities and outcomes available should their transplant patient be afflicted. It serves not only as a novel description of pVAD use in the transplant recipient, but also as an overview of treatment for mild to severe Takotsubo Syndrome all of which we have experienced at our centers amongst transplant patients, making it the largest series to date.

## Figures and Tables

**Table 1a:** (a) Literature review of Takotsubo Syndrome following abdominal transplant

Authors	# of patients	Sex	Age	Disease	Organ transplanted	Management	Outcome
Anders et al. [14]	One	Male	66	Laennec's cirrhosis	Liver	IABP on POD 2–23	Full recovery of cardiac function 40 days postop
Bedanova et al. [15]	One	Female	51	Autoimmune hepatitis and cirrhosis	Liver	IABP on POD 2–9	Full recovery of cardiac function 30 days postop
Chrapko et al. [16]	One	Female	46	Polycystic kidney disease	Kidney	Medical management. Diagnosed by TEE and ^123^I-mIBG myocardial uptake	Full recovery of cardiac function by 180 days postop
Eagle et al. [17]	One	Male	64	Laennec's cirrhosis	Liver	RCA vasospasm found on cardiac catheterization, apical ballooning persisted. Transvenous pacemaker placed. Delayed biliary reconstruction to the following day	Small subdural hematoma treated medically. Discharged to rehabilitation facility on POD 16
Gołębiewska et al. [18]	One	Female	68	Glomerulonephritis	Kidney	Medical management. Possible association with Calcineurin Inhibitor Toxicity.	Full recovery of cardiac function in 22 days post op
Harika et al. [8]	One	Male	52	Hemochromatosis and Laennec's cirrhosis	Liver	Medical Management with vasopressors	Full recovery of cardiac function in 18 days postop
Lee et al. [19]	One	Female	65	Nonalcoholic steatohepatitis with hepatocellular carcinoma	Liver	Medical management	Full recovery of cardiac function 6 weeks postop
Phillips et al. [20]	One	Female	60	Primary biliary cirrhosis, pulmonary embolus, renal failure, clostridium difficile colitis	Liver	Medical management with beta blockade and aspirin	Full recovery of cardiac function 16 days postop
Saner et al. [21]	1/2	Male	60	Hepatitis B cirrhosis	Liver	Medical Management with beta blockade.	Recovery, doing well 4 years after transplant
	2/2	Female	62	Hepatitis C cirrhosis	Liver	Medical management with vasopressors, hemodialysis	Death within 24 hours after transplant
Tachotti Pires et al. [22]	1/2	Female	33	Primary sclerosing cholangitis	Liver	Medical management initially with vasopressors, then ACEI and beta blockade	Recovery of EF on POD 10 though diagnosed with apical hypertrophic cardiomyopathy maintained on beta blockers
	2/2	Male	36	Budd-Chiari syndrome	Liver	Medical management with vasopressors	Recovery of cardiac function by POD 7 but found to have bacterial endocarditis, and expired 27 days postop
Tiwari and D'Attellis [23]	One	Female	45	Nonalcoholic steatohepatitis	Liver	Medical management with vasopressors. Delayed biliary reconstruction to the following day. IABP POD 2–5	Resolution in 12 days after transplant, though patient expired from hemorrhage
Vailas et al. [24]	One	Male	51	Trauma-inducing ESRD	Kidney	Medical management with vasopressors.	Full recovery of cardiac function 4 days postop, but attempt at stenting RAS led to loss of allograft on POD 56
Vachiat et al. [25]	One	Male	56	Laennec's and HCV cirrhosis	Liver	Left ventricular assist device	Full recovery of cardiac function by POD 25

IABP: intra-aortic balloon pump; POD: postoperative day; TTE: transthoracic echocardiogram; RCA: right coronary artery; ACEI: ace inhibitor; EF: ejection fraction; RAS: renal artery stenosis.

**Table 1b:** (b) Case demographics, management, and outcomes summary

Case	Sex	Age	Disease	Organ transplanted	Management	Outcome
Case 1	Female	45	IgA nephropathy	Kidney	Medical management	Full cardiac recovery POD 6
Case 2	Male	54	Laennec's cirrhosis	Liver	IABP	Near-total recovery of heart function at 3 mo
Case 3	Male	34	Laennec's cirrhosis	Liver	IABP, ICD	Partial recovery (EF 35%) at 5 mo
Case 4	Male	57	Laennec's and Hepatitis C cirrhosis	Liver	IABP	Death, POD2
Case 5	Female	46	Laennec's cirrhosis	Liver	IABP, ICD (preexisting)	Partial recovery (EF 35%) at 1 mo
Case 6	Male	57	Laennec's and Hepatitis C cirrhosis	Liver	TandemHeart	Full cardiac recovery 3 mo
Case 7	Male	66	Laennec's and Hepatitis C cirrhosis	Liver	TandemHeart	Full cardiac recovery 1 mo

IABP: intra-aortic balloon pump; POD: postoperative day; ICD: implantable cardioverter defibrillator; EF: ejection fraction.

## Abbreviations

CAD: Coronary artery disease
CVVHD: Continuous venovenous hemodialysis
EF: Ejection fraction
LV: Left ventricular
LVEF: Left ventricular ejection fraction
HCV: Hepatitis C virus
IABP: Intra-aortic balloon pump
MELD: Model for End Stage Liver
OLT: Orthotopic liver transplantation
pVAD: Percutaneous ventricular assist device
POD: Postoperative day
RVR: Rapid ventricular rate
TTE: Transthoracic echocardiography.
